# The role of Nod-like receptor protein 3 inflammasome activated by ion channels in multiple diseases

**DOI:** 10.1007/s11010-022-04602-1

**Published:** 2022-11-15

**Authors:** Xiaolin Xu, Xianli Wu, Gengyu Yue, Qimin An, Jun Lou, Xiaoxu Yang, Zhe Jin, Jianhong Ding, Yanxia Hu, Qian Du, Jingyu Xu, Rui Xie

**Affiliations:** 1 Department of Gastroenterology, Digestive Disease Hospital, Affiliated Hospital of Zunyi Medical University, 149 Dalian Road, Zunyi, 563003 Guizhou China; 2 The Collaborative Innovation Center of Tissue Damage Repair and Regeneration Medicine of Zunyi Medical University, Zunyi, China

**Keywords:** Inflammasome, Nod-like receptor protein 3, K^+^, Ca^2+^, Ion channel, Inflammatory diseases

## Abstract

The inflammasome is a multimeric protein complex located in the cytoplasm that is activated by many factors and subsequently promotes the release of proinflammatory factors such as interleukin (IL)-1β and IL-18, resulting in a series of inflammatory responses that ultimately lead to the occurrence of various diseases. The Nod-like receptor protein 3 (NLRP3) inflammasome is the most characteristic type and the most widely studied among many inflammasomes. Activation of the NLRP3 inflammasome is closely related to the occurrence of many diseases, such as Alzheimer's disease. At present, a large number of studies have focused on the mechanisms underlying the activation of the NLRP3 inflammasome. Plenty of articles have reported the activation of the NLRP3 inflammasome by various ions, such as K^+^ and Na^+^ reflux and Ca^2+^ influx. However, few articles have reviewed the effects of various ion channels on the activation of the NLRP3 inflammasome and the relationship between the diseases caused by these proteins. This article mainly summarizes the relationship between intracellular and extracellular ion activities and ion channels and the activation of the NLRP3 inflammasome. We also provide a general summary of the diseases of each system caused by NLRP3 activation. We hope that more research will provide options for the treatment of diseases driven by the NLRP3 inflammasome.

## Introduction

InflammasomeS are multimeric protein complexes that were first proposed by Jurg Tcholop in 2002, are assembled by intracytoplasmic pattern recognition receptors and are an important component of innate immunity [1]. The assembly of the inflammasome occurs in many cells, such as macrophages, dendritic cells, neutrophils and epithelial cells [2]. When the body is invaded by pathogenic microorganisms or injured by endogenous danger signals, pattern-recognition receptors (PRRs) recognize pathogen-associated molecular patterns (PAMPs) and danger-associated molecular patterns (DAMPs), induce the assembly of the inflammasome, and promote the release of the proinflammatory cytokines interleukin (IL)-1β and IL-18, the occurrence of an immune response and pyroptosis, which exerts significant positive effects on immune defenses against bacteria and viruses and the repair of damaged tissues. However, an increasing number of studies have suggested that inflammasome activation is closely related to many diseases [3–5]. Inflammasomes are classified according to the subcellular localization of the PRR. Nucleotide-binding and oligomerization domain Nod-like receptors, RIG-I-like receptors and AIM2-like receptors are located inside cells, and Toll-like receptors and C-type lectin receptors are located in the plasma membrane and endosome [6]. At present, many inflammasomes are known, but the most powerful and the most extensively studied inflammasome is the Nod-like receptor protein 3 (NLRP3) inflammasome; therefore, we chose it for this review.

The NLRP3 inflammasome belongs to the NLR protein family and is the most characteristic member. NLRP3 is composed of an N-terminal pyrin domain, a central nucleotide-binding domain and a C-terminal leucine-rich repeat domain [7]. The assembly of the NLRP3 inflammasome includes upstream sensor proteins (NOD-like receptors), adaptor proteins, apoptosis-related speck-like proteins, including apoptosis-associated speck-like protein containing a caspase recruit domain (ASC), and the downstream effector protein caspase-1[8]. Abnormal activation of the NLRP3 inflammasome is closely related to the occurrence and development of different diseases in various systems, such as Alzheimer's disease, coronary atherosclerosis and diabetes [9]. Increasingly, some ion-related cellular events have been shown to play an important role in the activation of the NLRP3 inflammasome, such as K^+^ and Cl^−^ efflux, Ca^2+^ mobilization, Na^+^ influx, and intracellular acidification [10, 11]. Although NLRP3 has been studied in many systems and many related reviews have been published, NLRP3-activated ion signaling requires greater work to refine and a detailed overview of the relationship between NLRP3 and intracellular and extracellular ions, ion channels, and diseases is currently unavailable. Hence, we will focus on this topic in our review. This review is expected to provide new targets for or insights into the study on the pathogenic mechanism of NLRP3 and treatment of NLRP3-related diseases.

## Cellular events related to NLRP3 activation

### The recognized signaling pathways of inflammasomes

**Fig. 1:**
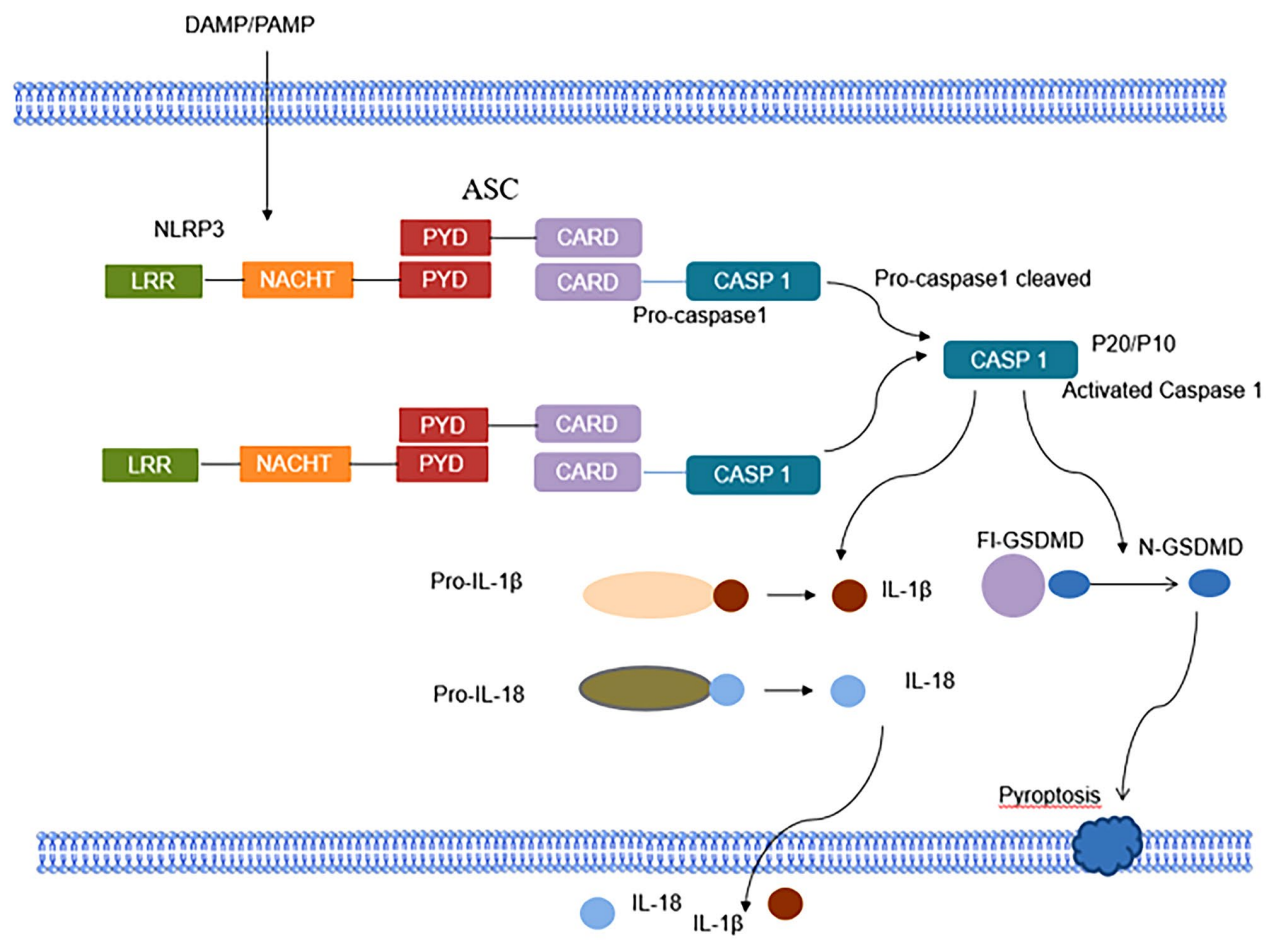
NLRP3 inflammasome activation. Upon activation of NLRP3 by PAMPs, DAMPs, and environmental stimuli, NLRP3 oligomerization results in clustering and presentation of the PYD domain, interacting with the PYD- and CARD-containing adapter ASC, whose CARD domain in turn recruits the CARD of procaspase-1. Pro-caspase-1 self-cleaves and forms active caspase-1 p10/p20 tetramers, which subsequently mature cytokine precursors such as pro-IL-1β and pro-IL-18 to IL-1β and IL-18, causing inflammation reaction. On the other hand, active caspase-1 promotes GSDMD cleavage leading to pyroptosis

**Fig. 2:**
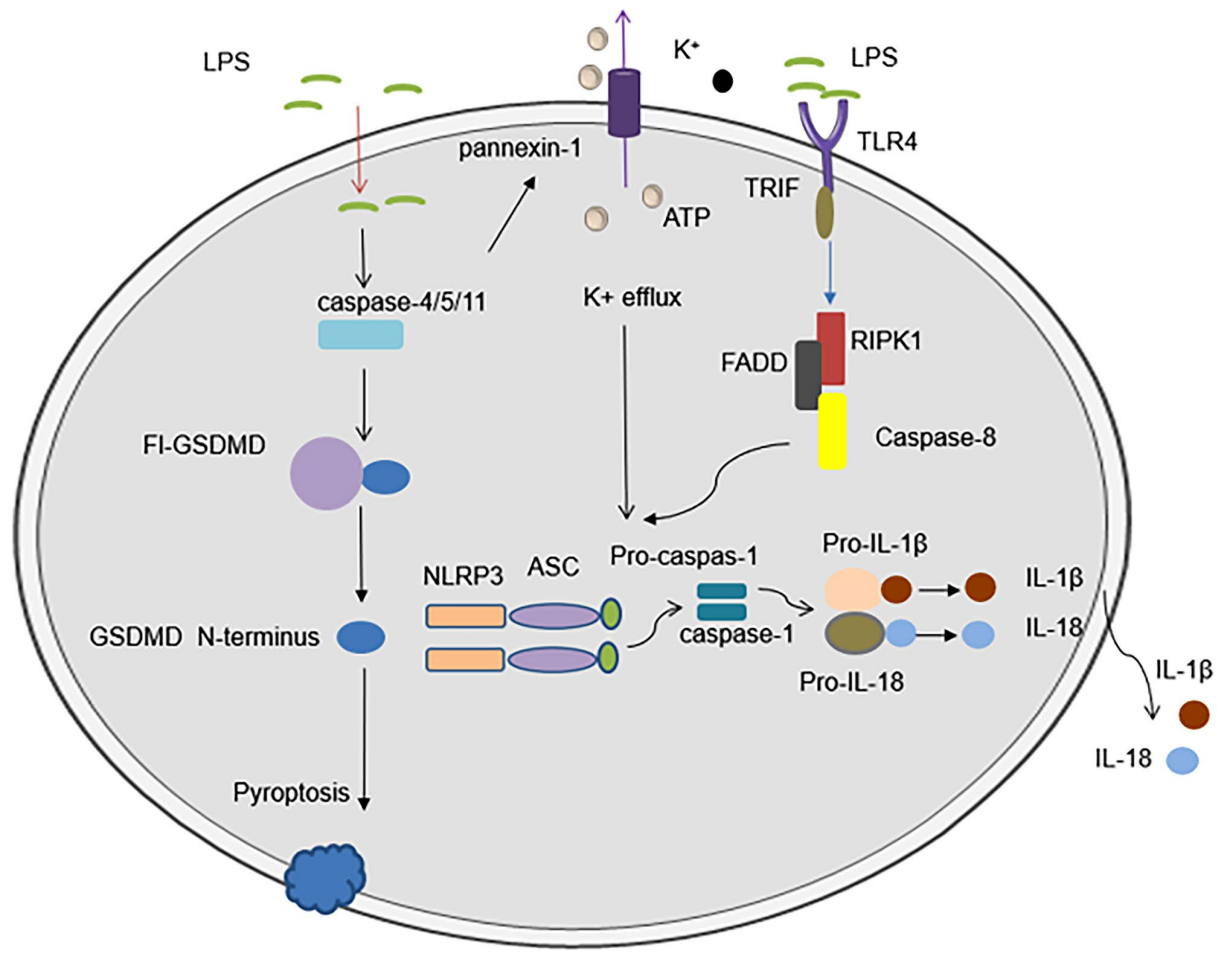
Non-canonical and alternative activation pathways of NLRP3. In the non-canonical activation pathway of the NLRP3 inflammasome, lipopolysaccharide (LPS) secreted by Gram-negative bacteria can promote GSDMD cleavage by interacting with caspase-4/5/11, thereby inducing pyroptosis, and, caspase- 4/5/11 can activate NLRP3 by activating an ATP-gated cation-selective channel that opens to trigger K^+^ efflux. (Fig. 2) In addition, in human monocytes, LPS can recognize TLR4 to activate NLRP3 through the TLR4-TRIF-RIPK1-FADD-CASP8 signaling pathway, termed the alternative activation pathway of NLRP3

The activation of NLRP3 inflammasome is divided into canonical activation pathway and non-canonical activation pathway. The initiation signal is usually recognized by Toll-like receptor (TLRs) ligands for some microbial molecules or by some cytokine ligands, and promotes the transcription of inflammasome-related components through the activation of nuclear factor-kappa B (NF-κB) and the post-translational modification of the NLRP3 inflammasome. [12]. DAMPs and PAMPs are the activation signals that trigger NLRP3 activation, and these components include extracellular ATP, bacterial pore-forming toxins, melanocin, and particulate matter (uric acid crystals, silica, etc.) [13]. The assembly of the inflammasome is through the interaction between the pyrin domain (PYD) of the ASC and the PYD of the NLR to recruit ASC to form ASC spots, which are then recruited through the caspase-activation and recruitment domain CARD-CARD interaction. Then, CARD domain in turn recruits the CARD of procaspase-1. Pro-caspase-1 clustering permits autocleavage and formation of the active caspase-1 p20/p10 tetramer, which promotes cleavage and maturation of downstream cytokines IL-1β and IL-18, ultimately they lead to a series of inflammatory responses [14, 15]. Besides, active caspase-1 can also cleavage of gasdermin D (GSDMD) induces pyroptosis, a novel form of cell death [4, 16]. On the other hand, in the non-canonical activation pathway of the NLRP3 inflammasome, lipopolysaccharide (LPS) secreted by Gram-negative bacteria can promote GSDMD cleavage by interacting with caspase-4/5/11, thereby inducing pyroptosis [17, 18] (Fig. 1). Furthermore, caspase-4/5/11 can activate NLRP3 by activating an ATP-gated cation-selective channel that can open to trigger K^+^ efflux [19] (Fig. 2). In addition, in human monocytes, LPS can recognize TLR4 to activate NLRP3 through the TLR4-TRIF-RIPK1-FADD-CASP8 signaling pathway, termed the alternative activation pathway of NLRP3 (Fig. 2) [20].

### Ion regulatory mechanism during NLRP3 activation

#### K^+^ efflux is a recognized upstream signal of NLRP3 activation

**Fig. 3:**
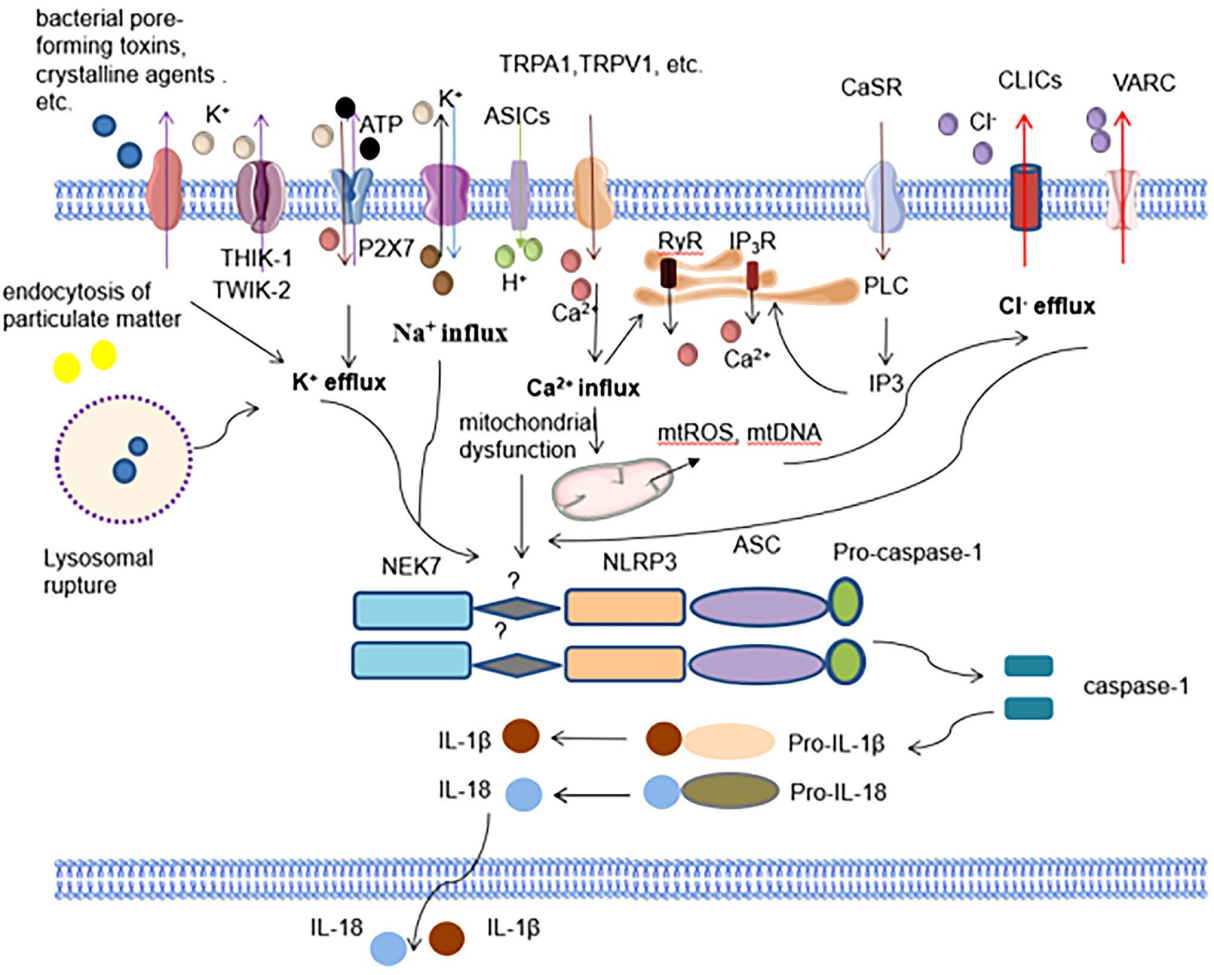
Intracellular ion signaling associated with NLRP3 activation. K^+^ efflux is a recognized upstream signal of NLRP3 activation. NLRP3 agonists induce the opening of K^+^ channels such as P2X7 receptors, THIK-1, TWIK-2, etc., mediate potassium efflux, and promote NLRP3 inflammasome activation. Opening of ion channels of the transient receptor potential family, calcium-sensing receptors catalyze the production of inositol-1,4,5-triphosphates by phospholipase C, induce the release of calcium ions from the IP3R of the endoplasmic reticulum and the membrane attack complex-induced Ca2 + Influx, through activation of RyR to mediate calcium release from endoplasmic reticulum storage, and P2X7 receptors to mediate trace Ca^2+^ influx, excessive or sustained mitochondrial Ca^2+^ uptake can lead to mitochondrial damage, release mtROS and mtDNA into the cytoplasm, ROS is a common signal for the activation of the NLRP3 inflammasome, and the mechanism needs to be further explored. In addition, chloride efflux mediated by chloride channels CLICs and VARC, Na^+^ influx mediated by NHA channel opening, reduction of cellular pH induced by acid-sensing channel ASICs, and lysosomal disruption by granule endocytosis are upstream of NLRP3 activation cell signaling

Studies have shown that a decrease in intracellular K^+^ levels is an important stimulus that activates NLRP3 through a mechanism induced by ATP and other DAMPs [21, 22]. V P E´trilli et al. found that high extracellular potassium levels inhibit the activation of NLRP3 in human monocytes and that a decreased intracellular potassium concentration triggers the activation of the NLRP3 inflammasome [23]. Raúl Muñoz-Planillo and other scholars aimed to further determine the role of K^+^ efflux in the activation of the NLRP3 inflammasome and proved that the activation of the NLRP3 inflammasome causes a decrease in intracellular K^+^ concentration. They also found that ASC oligomerization, also called ASC aggregation into ASC speck macromolecules during inflammasome activation, is suppressed by high extracellular K^+^ concentrations, indicating that K^+^ regulates NLRP3 inflammasome activation [24]. P2X7, a kind of P2 receptors can be activated by ATP aggregation, which is required for ATP aggregation-induced activation of NLRP3 [25]. In recent years it has been found that P2X7 may partially regulate K^+^ currents through the two-pore domain K^+^ channels, such as Pore Domain Halothane-Inhibited Potassium Channel 1 (THIK-1) and Two-pore domain Weak Inwardly rectifying K^+^ channel 2 (TWIK-2) [26] (Fig. 3). In addition, the K^+^ efflux caused by some microbial toxins and the destruction of the cell membrane is also a pathway activating the NLRP3 inflammasome [27, 28]. According to recent studies, K^+^ efflux promotes the activation of the NLRP3 inflammasome through a mechanism that may be induced by mitochondrial damage and mitochondria ROS (mtROS) production [10, 29]. In addition, Yuhua Chen et al. reversed neuroinflammation caused by brain injury by knocking down NIMA-related kinase 7 (NEK7). This study further proved that NEK7 is useful as a modulator by regulating the interaction of NEK7–NLRP3, and thus the activation of the NLRP3 inflammasome is mediated by K^+^ efflux [12, 21, 30, 31]. Furthermore, the interaction between NLRP3-NEK7 may not be sufficient to activate NLRP3, which may also be required to convert a central nucleotide-binding and oligomerization from an inactive to an active conformation because of NLRP3 oligomerization. This conformational transition likely requires ATP binding and other unknown allosteric triggers [32] (Fig. 3).

#### Increased intracellular Ca^2+^ levels promote NLRP3 activation

As an indispensable second messenger in many cellular processes, Ca^2+^ plays an important role in the activation of the NLRP3 inflammasome. Extracellular ATP promotes Ca^2+^ influx through the P2X7 receptor, which induces the activation of the NLRP3 inflammasome, but this conclusion remains controversial and more evidence is needed to prove it [33–35]. Increasing levels of extracellular calcium ions stimulate G protein-coupled calcium-sensitive receptors to catalyze the production of inositol-1,4,5-triphosphate via phospholipase C, induce Ca^2+^ release from endoplasmic reticulum stores and then activate the NLRP3 inflammasome [36, 37]. Ca^2+^ influx induced by the membrane attack complex subsequently promotes the release of calcium stored in the endoplasmic reticulum by activating the ryanodine receptor (RyR) or inositol 1,4,5-trisphosphate receptor (IP3R), which will further increase the intracellular Ca^2+^ concentration and cause mitochondrial dysfunction through mitochondrial calcium transporters, promoting the activation of the NLRP3 inflammasome[38]. Hidenori Ichijo et al. found that the destruction of lysosomes regulates the activation of NLRP3 inflammasomes and promoting ASC oligomerization [39]. In addition, some inflammasome activators mediate IL-1β release by promoting the opening of a transient receptor potential (TRP) family of ion channels, including TRPM2, TRPM8, TRPM7, and TRPV2. The fluctuation of Ca^2+^ and Mg^2+^ concentrations promote the phosphorylation of TAK1, which in turn activates the NLRP3 inflammasome [13, 40, 41]. Importantly, studies have shown that excessive or sustained Ca ^2+^ uptake leads to mitochondrial damage characterized by increased mtROS production, a shift in mitochondrial permeability, and ultimately mitochondrial rupture, releasing mtROS and mitochondria DNA (mtDNA) into the cytoplasm, demonstrating that Ca ^2 +^ Stimulation of mobilization leads to mitochondrial damage to activate the NLRP3 inflammasome, and mtROS is required for the NLRP3 inflammasome in response to LPS and ATP activation [42, 43] (Fig. 3). Most NLRP3 agonists can induce ROS production, a common signal of NLRP3 inflammasome activation [44, 45]. However, in-depth studies are needed to determine the role and mechanism of ROS on NLRP3 inflammasome activation.

#### Cl^−^ efflux and NLRP3 inflammasome activation

Cl^−^ efflux has been shown to be another regulatory pathway that regulates NLRP3 activation. A decrease in intracellular chloride ions promotes the ATP-induced processing and secretion of IL-1β [46]. Hypotonicity induces IL-1β release by activating the NLRP3 inflammasome; hypertonicity exerts its effect through the mechanism of regulatory cell volume reduction (RVD) achieved by reducing the concentrations of K^+^ and Cl^−^ in the cell [47]. Significant cell expansion occurs in macrophages stimulated with LPS-ATP, and this response is controlled by the synergistic effects of K^+^ and Cl^−^ [41]. The inhibitory effects of high concentrations of extracellular NaCl and chloride ion inhibitors also indicate that chloride plays a role in the activation of the NLRP3 inflammasome [48]. Recent studies have found that intracellular Cl^–^ concentration at 75 mM indirectly regulates NLRP3 and the pyrin domain of caspase 1 (CASP1) with maximum expression and activity, as well as greater ROS activity, while PYD and CARD domain-containing (PYCARD/ASC)) expression remained constant from 0 to 125 mM Cl^–^. Cl^–^ can stimulate the secretion of mature IL-1β through regulation, which in turn upregulates ROS, caspase-1, NLRP3 and IL-1β itself through autocrine signaling [49]. At the same time, a recent study determined that (leucine-rich repeat-containing protein 8A) LRRC8A, an important component of the volume-regulated anion channel (VRAC), is required for hypotonic induction of NLRP3 inflammasome activation. Therefore, other chloride channels also regulate the activation of the NLRP3 inflammasome [50]. In addition, CLIC1 and CLIC4, which are intracellular chloride channel proteins (CLICs), are involved in LPS-induced IL-1β activation and modulate NLRP3 activation [48]. CLIC-dependent chloride efflux is located downstream of the potassium efflux-mitochondrial ROS axis and alters the activation of the NLRP3 inflammasome by regulating the NEK7-NLRP3 interaction [29] (Fig. 3).

#### Na^+^ influx participates in NLRP3 activation

Na^+^ influx plays a role in NLRP3 activation, but it is not a necessary condition for NLRP3 activation (Fig. 2). In one study, gramicidin or other NLRP3 stimulators required a certain concentration of extracellular Na + to activate NLRP3. The authors found that this process also caused a decrease in K^+^ levels in the cell. Na^+^ influx may regulate NLRP3 activation by reducing the decrease in K^+^ in cells [24]. However, a large amount of Na^+^ released from mature endosomes and lysosomes induced by monosodium urate (MSU) crystals triggers the influx of water to reduce the intracellular K^+^ concentration rather than causing net cytoplasmic K^+^ loss [51]. In addition, the activation of the P2X7 receptor induced by the accumulation of large amounts of ATP promotes Na^+^ influx and subsequently induces the activation of the NLRP3 inflammasome [22]. Based on this evidence, Na^+^ influx exerts a certain effect on activating inflammasomes. However, the specific activation mechanism must be further clarified.

#### Intracellular pH and NLRP3 inflammasome activation

The dynamic balance of pH inside and outside the cell is necessary to maintain the various biochemical reactions in the cell, and a decrease in pH exerts a certain effect on promoting the occurrence of tissue inflammation. Under acidic conditions, the low pH induces NLRP3 activation and IL-1β release through the K^+^ efflux mechanism, as well as the secretion of active cathepsin B [52]. In addition, after the virus is engulfed by endosomes and enters the cell, it fuses to form a membrane pore in an acidic environment, which promotes virus replication, causes infection, and activates the inflammasome [53] (Fig. 2). It was recently found that in influenza A virus infection, the M2 proton channel protein is activated under acidic conditions of pH 6 and causes the fusion of the endosomal membrane and the viral membrane, resulting in the release of viral genetic material into the cytoplasm and regulation of Golgi dispersion, Recruitment and activation of the NLRP3 inflammasome leads to the production of IL-1β, which may be the induction of influenza A pathogenesis[54]. Furthermore, extracellular lactate was found to regulate intercellular reactive oxygen species (ROS) levels through proton-gated channel subfamilies acid-sensitive ion channel 1 (ASIC1) and ASIC3. ROS promotes NLRP3 inflammasome activation and IL-1β release by activating the NF-κB signaling pathway. Therefore, ASIC is expected to be a potential target for future treatment of NLRP3 inflammasome activation-related diseases [55] (Fig. 3).

## Inflammasomes, ion channels and diseases

### Parkinson's disease (PD)

Parkinson's disease is a neuroinflammatory disease characterized by loss of dopamine function. α-Synuclein (αSyn) in microglia mediates NLRP3 inflammasome activation to induce IL-1β secretion, and pyroptosis induced by αSyn damages dopamine neurons and causes Parkinson's disease [56, 57]. The ATP-sensitive K^+^ (K-ATP) channel is a member of the inward rectifying channels and consists of four pore-forming Kir6.x (Kir6.1 or Kir6.2) subunits and four regulatory sulfonate urea receptor (SUR1 or SUR2) subunits [58]. Studies have found that Kir6.1 (one of the ATP-sensitive potassium (K-ATP) channel subunits) deletion inhibits astrocyte phagocytosis by inducing mitochondrial dysfunction and mitochondrial ROS production, which contribute to the excessive activation of the NLRP3 inflammasome and the production of proinflammatory factors, thereby inducing the occurrence of PD. Thus, the Kir6.1/K-ATP channel expressed in astrocytes may be a target for preventing the degeneration of DA neurons in patients with PD and treating PD [59]. Besides, calcium ions released from the endoplasmic reticulum induce NLRP3 inflammasome assembly, as well as increased osmotic pressure in glial cells, active caspase-1 cleaves GSDMD to obtain GSDMD-CT (C-terminal fragment) and GSDMD-NT (N-terminal fragment), the latter one promotes the formation of membrane pores and regulate cell pyroptosis, thereby promoting neuroinflammation. Researchers found that THIK-1 is required for NLRP3-dependent Caspase-1 activation and IL-1β release in response to ATP. By blocking THIK-1, it inhibits the release of Pro-inflammatory cytokine il-1β from the activated microglia, which suggests that THIK-1 may be a therapeutic target for Nervous system inflammation diseases [26, 60].

### Alzheimer's disease (AD)

The activation and aggregation of microglia induced by the deposition of the fibrillary peptide amyloid-β (Aβ) is a neuronal abnormality that is the basis of dementia and is an important pathological factor leading to the occurrence of Alzheimer's disease [57]. Aβ induces caspase-1 activation and the release of mature IL-1β by activating NLRP3, causing neuroinflammation and neurotoxicity [61]. The nonsteroidal anti-inflammatory drug fenamate inhibits the NLRP3 inflammasome by blocking a Cl^−^ channel on the plasma membrane called the VRAC Ticagrelor (Table 1). This drug inhibits Alzheimer’s disease-related cognitive impairment in a rodent model, which provides an option for the treatment of NLRP3-related inflammatory diseases [62]. In addition, knockout of mouse microglia Na^+^-K^+^-2Cl^−^ cotransporter (NKCC1) leads to the initiation of the NLRP3 inflammasome and increases the production of interleukin 1β (IL-1β), thereby predisposing microglia to excessive inflammatory response, showing significantly increased brain damage, inflammation, cerebral edema, and worse neurological outcomes [63]. This suggests that the Kir6.1/K-ATP and Na^+^-K^+^-2Cl^−^ channel of astrocytes may be a target for preventing the degeneration of dopamine neurons in PD and treating PD.

**Table 1:** Ions and possible treatments for NLRP3 inflammasome activation-driven diseases

Ion	Ion channel	Disease	Possible method	References
K^+^	Kir6.1(K-ATP) channel	①PD ②T2DM		[59] [94]
	THIK-1	neurological disease		[26]
	NKCC1	PD		[63]
	Kir6.2	liver damage	①Iptakalim ②Glibenclamide	[81]
	KCa1.1,KCa3.1(Calcium-activated potassium channels)	rheumatoid arthritis		[84]
	Kv4.2	heart failure	MCC950	[76]
Ca^2+^	transient potential receptor (TRPA1、TRPV1)	pneumonia	A967079 AMG9810	[66]
	TRPM2	T2DM		[35]
	Calmodulin-dependent protein kinase	heart failure	Engligliflozin	[70, 71]
	TRPML1	Kidney damage		[90]
	NCLX (Na^+^-Ca^2+^ exchanger)	T2DM		[95]
Cl^−^	Volume-regulated anion channel	Alzheimer's disease	Fenamate	[62]
	ANO1(Calcium ion activated chloride channel)	myocardial ischemia		[68]
	CLICs	heart failure	Ticagrelor	[78]
Na^+^	NHE(Na^+^-H^+^ exchanger)	heart failure	Engligliflozin	[72]
	Na^+^-K^+^ATPase(NKA)	nephritis		[89]
H +	ASCLs (acid-sensitive ion channel)1a	rheumatoid arthritis		[86]

Changes in the concentrations of Na^+^, K^+^, Ca^2+^, Cl^−^ and H^+^ in cells may activate NLRP3 and drive the occurrence of different diseases. Different ion channels are involved in the occurrence of these diseases, and damage to the function or changes in the states of these ion channels may cause different diseases. Therefore, therapeutic methods targeting a certain ion channel may be useful to treat a certain disease

### Pneumonia

PM2.5 is a major factor affecting people's health due to urbanization. When PM2.5 enters the lungs from the respiratory tract, it may cause pneumonia, airway hyperresponsiveness and even asthma [64]. PM2.5 enters cells through a variety of phagocytosis processes and release cathepsin B to produce ROS and mediate K^+^ efflux, which in turn activate the NLRP3 inflammasome and eventually lead to lung inflammation and pulmonary fibrosis [65]. Similarly, in some experiments, an intranasal instillation of PM2.5 in mice caused lung inflammation, airway hyperresponsiveness, and oxidative stress in mice, because instilled PM2.5 increases the levels of H_2_O_2_ and mtROS and subsequently directly activates TRPV1 and TRPA1. Then, Ca^2+^ influx mediated by the opening of TRPV1 and TRPA1 activate the NLRP3 inflammasome, and the increase in mtROS levels also directly induces the activation of the NLRP3 inflammasome. The authors also found that the use of the TRPV1 antagonist AMG9810 and the TRPA1 antagonist A967079 alone or in combination alleviates pneumonia in mice induced by these processes. Inhibition of TRPA1 alone or TRPV1 and TRPA1 in combination may be more effective than inhibition of TRPV1 alone in treating PM2.5-induced lung injury [66]. Deletion of the *Kcnk6* gene (encoding TWIK2) suppressed NLRP3 activation in macrophages and suppressed sepsis-induced lung inflammation. Adoptive transfer of Kcnk6^−/−^ macrophages into the mouse airways after macrophage depletion also prevents inflammatory lung injury [21].

### Heart failure

Studies have shown that the necrotic myocardium after myocardial infarction can act as DAMP to induce the assembly of NLRP3 inflammasome, causing cardiac inflammation and accelerating heart failure. Therefore, the targeted therapy of NLRP3 inflammasome may be a feasible strategy to reduce the area of myocardial infarction and prevent heart failure after acute myocardial infarction [67]. Some studies found that the upregulation of miR-144-3p significantly reduced myocardial ischemia/reperfusion injury (MIRI) in vivo and in vitro. They further proved that Ca^2+^-activated Cl^−^ channels encoded by anoctamin-1 (ANO1) is the target gene of miR-144- 3p. Targeting miR-144-3p / ANO1 can inhibit the activation of NLRP3 inflammasome inflammatory signal in myocardial cells. It provides new insights for targeted therapy of myocardial ischemia [68]. Studies have shown that the overexpression of Ca^2+^ /calmodulin-dependent protein kinase IIδ (CMCaMKIIδ) can cause heart failure and contribute to the expression of inflammatory genes [69–71]. In recent years, based on the occurrence of arrhythmia and oxidative stress are all related to the increase of the concentration of Na^+^ and Ca^2+^ in myocardial cells. Long-term inhibition of Na^+^/H^+^ exchanger (NHE) can prevent or reduce heart failure [72]. A new antidiabetic drug empagliflzin, which is a selective inhibitor of renal proximal tubule sodium-dependent glucose transporter 2 (SGLT2), can inhibit cardiac NHE and reduce CaMKII activity and CaMKII-dependent sarcoplasmic reticulum calcium ion leakage. Thereby reduce cardiac cytoplasm [Na^+^] and [Ca^2+^] and increase cardiomyocyte mitochondria [Ca^2+^], which can inhibit the initiation and activation of NLRP3 inflammasome, thus have a direct anti-inflammatory effect on the heart and effectively improve the prognosis of heart failure in diabetic patients [73–75]. The study found that administration of MCC950, a specific NLRP3 inflammasome inhibitor, could inhibit the NLRP3 inflammasome by upregulating the expression of ion channel proteins (Kv4.2, KChIP2 and Cav1.2) in mice with heart failure, thereby improving the vulnerability of ventricular arrhythmias caused by heart failure [76]. In addition, chloride channel blockers can inhibit ROS-promoted CLIC-induced chloride plasma membrane translocation in cardiomyocytes and restore NLRP3-mediated cardiomyocyte pyroptosis [77]. In addition, ticagrelor is an oral P2Y12 receptor antagonist, which can rapidly and robustly inhibit the activation of NLRP3 inflammasome in peripheral blood mononuclear cells of patients with acute coronary syndrome by degrading chloride intracellular channel proteins (CLICs) and blocking the translocation of CLICs to the plasma membrane [78]. (Table 1).

### Liver damage

Based on the role of NLRP3 in obesity-related metabolic syndrome, experiments have shown that saturated fatty acids activate the NLRP3 inflammasome by inducing the destruction of NKA caused by the accumulation of saturated phosphatidylcholine and the loss of plasma membrane fluidity. Therefore, cotreatment with unsaturated fatty acids may represent a new treatment approach for reducing obesity-related inflammation, such as nonalcoholic liver cirrhosis and insulin resistance [79]. K-ATP channels are involved in the regulation of many cellular activities as metabolic sensors [80]. The opening of the Kir6.2/K-ATP channel protects mice from liver damage caused by LPS-induced activation of the NLRP3 inflammasome, continuous high levels of IL1β, IL-18, and TNF-α and excessive endoplasmic reticulum stress and autophagy of liver cells. Therefore, the new K-ATP channel opener iptakalim may exert a potential therapeutic effect on protecting against liver injury [81]. In contrast, studies have shown that low-dose glibenclamide (a type 2 diabetes drug that inhibits K-ATP channels in pancreatic β cells) downregulates TGF-β1, NLRP3, ASC, TGF-β1, NLRP3, and ASC expression induced by thioacetamide (Table 1). The expression of caspase-1 and IL-1β and upregulation of catalase resist thioacetamide-induced liver damage, which exerts a certain protective effect on the liver. However, appropriate doses of this drug and in vitro and in vivo experiments are needed before future clinical applications [82]. In general, approaches targeting ion channels related to the activation of the NLRP3 inflammasome may provide new insights into the treatment of liver inflammation.

### Rheumatoid arthritis (RA)

RA is a common chronic autoimmune disease that usually manifests as symmetrical and aggressive joint inflammation of multiple small joints. Studies have found that the NLRP3 inflammasome is highly activated in the synovium of patients with RA and collagen-induced rheumatoid arthritis model mice and that NLRP3 activation plays an important role in the pathogenesis of RA [83]. Researchers have found that the Ca^2+^-activated K^+^ channels KCa1.1 and KCa3.1 promote the occurrence of autoimmune diseases. One of the mechanisms underlying this event is the activation of NLRP3. Hydroxychloroquine HCQ, an ion channel inhibitor, inhibits the K^+^ channel activated by Ca^2+^, which is a strategy for the treatment of RA [84]. The expression of calcium-sensing receptor (CaSR) is increased in monocytes and locally damaged joints of patients with RA, and the activation of the NLRP3 inflammasome mediated by CaSR promotes the occurrence of RA. Therefore, inhibition of CaSR is also a strategy for the treatment of RA [85]. In addition, ASIC1a is an extracellular H^+^-activated cation channel that mainly affects the permeability of Na^+^ and Ca^2+^, which upregulate the NLRP3 inflammasome and the expression of proinflammatory factors to induce pyrolysis of chondrocytes in arthritic rats [86]. These discoveries provide a new direction for studying the mechanism of RA.

### Kidney damage

Uric acid (UA) crystals are one factor stimulating NLRP3 activation. Soluble UA increases NLRP3 expression in proximal renal tubular epithelial cells in a TLR4-dependent manner and promotes caspase-1 activation and the production of IL-1β and intercellular adhesion molecule 1, ultimately inducing innate immunity in proximal tubular epithelial cells of the kidney [87]. High UA levels also induce NLRP3 activation through ROS activation and K^+^ efflux to cause vascular endothelial cell damage [88]. Investigators have shown that damage to the basolateral Na^+^–K^+^–ATPase (NKA) leads to protein tyrosine kinase binding and dissociation of NKA, leading to NLRP3 activation, IL-1β upregulation, and renal inflammation. In addition, the expression of NKAα1 significantly reduces UA-induced ROS generation and reduces early-onset apoptosis, but has no effect on late apoptosis. Reversing UA accumulation leads to a decrease in the mitochondrial membrane potential and reduces mitochondrial dysfunction [89]. Based on these results, NKA exerts a protective effect on kidney damage caused by UA and provides a new insight into strategies for protecting the kidney. Hyperhomocysteinemia (hHcy)-induced podocyte NLRP3 inflammasome activation is an initiating event in glomerulonephritis. Hcy inhibits the transient receptor potential mucolipin 1 (TRPML1) channel activity in lysosomes by enhancing ROS generated by NADPH oxidase, resulting in reduced lysosome-multivesicular bodies (MVB) interactions and more exosome release in podocytes. Exosome secretion may be the pathogenic mechanism mediating the release of inflammatory cytokines produced by the NLRP3 inflammasome in podocytes. Targeting the TRPML1 channel provides a new therapeutic strategy for attenuating podocyte-derived inflammatory exosome release and consequent glomerular inflammation [90].

### Type 2 diabetes

Sufficient research evidence shows that type 2 diabetes is related to the chronic inflammatory response mediated by monocyte activation. In particular, IL-1β released by the activation of the NLRP3 inflammasome reduces tyrosine phosphorylation, and the negatively regulation of insulin receptor substrate 1 mRNA expression directly inhibits the insulin signaling pathway, leading to insulin resistance and type 2 diabetes mellitus (T2DM) [91, 92]. As mentioned above, K^+^ efflux and Na^+^ influx regulate the activation of the NLRP3 inflammasome. Similarly, experiments have shown that rats fed a high-salt diet exhibit increased oxidative stress followed by the activation of the NLRP3 inflammasome to induce insulin resistance, while potassium supplementation improves insulin resistance [93]. Thus, determining the role of NLRP3-activating related ion channels in the development of diabetes is important for improving insulin resistance. The K-ATP channel Kir6.1 is potentially useful as a negative regulator of the NLRP3 inflammasome and insulin resistance, and it is a very promising target for the treatment of diabetes [94] (Table 1). In addition, some experiments have found that hyperglycemia (30 mM glucose for 48 h) induces the activation of NADPH oxidase through TRPM2 channel-mediated Ca^2+^ influx in monocytes, which contributes to ROS generation and thioredoxin-interacting protein -mediated activation of the NLRP3 inflammasome. Thus, TRPM2 may represent a new target for ameliorating T2DM caused by hyperglycemia-induced oxidative stress and subsequent NLRP3 inflammasome activation [35]. Moreover, silencing the expression of the mitochondrial Na^+^/Ca^2+^ exchanger (NCLX) will hinder mitochondrial Ca^2+^ efflux that promotes ROS generation and mitochondrial damage, which finally results in the activation of endothelial cell apoptosis. Therefore, NCLX provides new insights into the mechanism of diabetic vascular disease and may also provide new strategies for the treatment of diabetes and its vascular complications [95].

## Conclusions and prospects

The NLRP3 inflammasome plays an important role in the inflammatory response and immune defenses and is closely related to the occurrence and development of many diseases. The mechanism underlying inflammasome activation has always been the focus of discussion, and changes in the plasma contents of K^+^, Ca^2+^, and Cl^−^ in cells are different cellular signals that modulate the activation of the NLRP3 inflammasome. In recent years, further studies have found that a variety of NLRP3 inflammasome activation-induced diseases involve different ion channels. Based on this knowledge, some treatment strategies have been proposed for diseases associated with abnormal activation of NLRP3. Although the mechanisms regulating these ions and the roles of ion channels are now understood, they are not comprehensive, the relationship between various ion signaling pathways is not sufficiently clear, and the other ion channels involved in NLRP3 inflammasome-induced diseases have not been sufficiently clarified. Therefore, more effort is needed to obtain additional information. For example, the unified activation pathway of NLRP3, calcium ion signaling between the endoplasmic reticulum and mitochondria, and the mechanism of ROS on NLRP3 inflammasome activation need to be further studied.

## Data Availability

Not applicable.

## Abbreviations

NLRP3: Nod-like receptor protein 3
PRRs: Pattern-recognition receptors
PAMPs: Pathogen-associated molecular patterns
DAMPs: Danger-associated molecular patterns
ASC: Apoptosis-associated speck-like protein containing a caspase recruit domain
TLRs: Toll-like receptors
LPS: Lipopolysaccharide
NF-κB: Nuclear factor-kappa B
PYD: Pyrin domain
CARD: Caspase-activation and recruitment domain
GSDMD: Gasdermin D
THIK-1: Pore domain halothane-inhibited potassium channel 1
TWIK-2: Two-pore domain weak inwardly rectifying K^+^ channel 2
NEK7: NIMA-related kinase 7
RyR: Ryanodine receptor
IP3R: Inositol 1,4,5-trisphosphate receptor
RVD: Cell volume reduction
VRAC: Volume-regulated anion channel
TRP: Transient receptor potential
mtROS: Mitochondria ROS
mtDNA: Mitochondria DNA
MSU: Monosodium urate
ASIC: Acid-sensitive ion channel
PD: Parkinson's disease
K-ATP channel: ATP-sensitive K + channel
SUR: Sulfonate urea receptor
AD: Alzheimer's disease
Aβ: Amyloid-β
ANO1: Ca^2+^-activated Cl^−^ channels encoded by anoctamin-1
NHE: Na^+^/H^+^ exchanger
RA: Rheumatoid arthritis
CaSR: Calcium-sensing receptor
SGLT2: Sodium-dependent glucose transporter 2
UA: Uric acid
NKA: Na^+^–K^+^–ATPase
T2DM: Type 2 diabetes mellitus
NCLX: Na^+^/Ca^2+^ exchanger
CASP1: Caspase 1
